# Œsculapian Society

**Published:** 1856-04

**Authors:** 


### EDITORIAL.

*Œsculapian Society.*

We learn from Dr. C. M. Hamilton, of Palestine, Illinois, that the next meeting of this Society will be held at York, Clark County, on the last Wednesday in May next. The Œsculapian Society is one of the most active and useful Medical Societies in the West. The readers of this Journal have had many good articles from its members, and we trust they may yet have many more.

The article alluded to by Dr. Hamilton, will be thankfully received.

